# Advanced bleeding control in combat casualty care: An international, expert-based Delphi consensus

**DOI:** 10.1097/TA.0000000000003525

**Published:** 2022-01-21

**Authors:** Suzanne M. Vrancken, Boudewijn L.S. Borger van der Burg, Joseph J. DuBose, Jacob J. Glaser, Tal M. Hörer, Rigo Hoencamp

**Affiliations:** From the Department of Surgery (S.M.V., B.L.S.B.v.d.B., R.H.), Alrijne Hospital, Leiderdorp, the Netherlands; Trauma Research Unit, Department of Surgery (S.M.V., R.H.), Erasmus MC, University Medical Center Rotterdam, Rotterdam, the Netherlands; R Adams Cowley Shock Trauma Center (J.J.D.), University of Maryland, Baltimore, Maryland; Naval Medical Research Unit San Antonio (J.J.G.), JBSA-Ft. Sam Houston, Texas; San Antonio Military Medical Center (J.J.G.), JBSA-Ft. Sam Houston, Texas; Department of Cardiothoracic and Vascular Surgery (T.M.H.), Faculty of Medicine and Health, Örebro University Hospital, Örebro University, Örebro, Sweden; Department of Surgery, Faculty of Medicine and Health (T.M.H.), Örebro University Hospital, Örebro University, Örebro, Sweden; Defense Healthcare Organization, Ministry of Defense (R.H.), Utrecht, the Netherlands; and Department of Surgery, Leiden University Medical Centre (R.H.), Leiden, the Netherlands.

**Keywords:** Hemorrhage control, Delphi consensus, combat casualties, REBOA, guidelines

## Abstract

Hemorrhage from truncal and junctional injuries is responsible for the vast majority of potentially survivable deaths in combat casualties, causing most of its fatalities in the prehospital arena. Optimizing the deployment of the advanced bleeding control modalities required for the management of these injuries is essential to improve the survival of severely injured casualties. This study aimed to establish consensus on the optimal use and implementation of advanced bleeding control modalities in combat casualty care.

**METHODS:**

A Delphi method consisting of three rounds was used. An international expert panel of military physicians was selected by the researchers to complete the Delphi surveys. Consensus was reached if 70% or greater of respondents agreed and if 70% or greater responded.

**RESULTS:**

Thirty-two experts from 10 different nations commenced the process and reached consensus on which bleeding control modalities should be part of the standard equipment, that these modalities should be available at all levels of care, that only trained physicians should be allowed to apply invasive bleeding control modalities, but all medical and nonmedical personnel should be allowed to apply noninvasive bleeding control modalities, and on the training requirements for providers. Consensus was also reached on the necessity of international registries and guidelines, and on certain indications and contraindications for resuscitative endovascular balloon occlusion of the aorta (REBOA) in military environments. No consensus was reached on the role of a wound clamp in military settings and the indications for REBOA in patients with chest trauma, penetrating axillary injury or penetrating neck injury in combination with thoracoabdominal injuries.

**CONCLUSION:**

Consensus was reached on the contents of a standard bleeding control toolbox, where it should be available, providers and training requirements, international registries and guidelines, and potential indications for REBOA in military environments.

## Abstract 2

Consensus on Advanced Bleeding Control in combat casualty care: an international, expert-based Delphi consensus study on the optimal implementation and use of advanced bleeding control techniques in military environments.#combat casualty care #vascular trauma #REBOA.

## Graphical abstract

**Figure:**
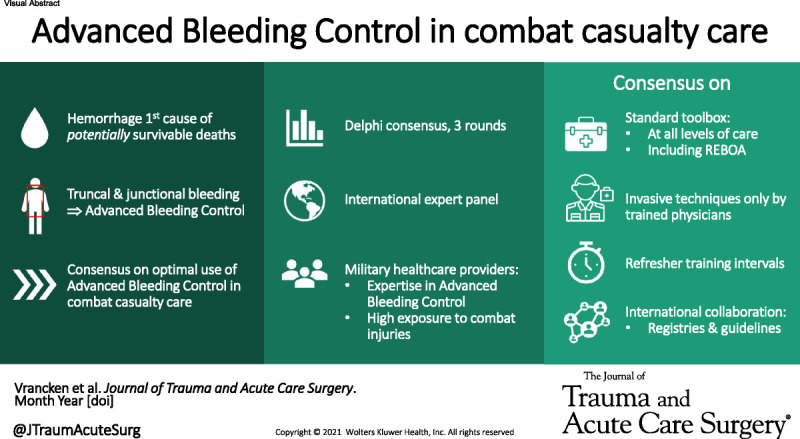

Armed conflicts have been prevalent throughout human history, inflicting large numbers of casualties. Over the last two decades, advances in military trauma systems contributed to a significant improvement in the survival of these casualties.^1,2^ Since 2003, increasing emphasis has been placed on rapid evacuation from the point of injury (POI) to surgical care through the tiered medical support echelons (Box 1), including improvements in POI and en-route care to provide advanced medical care earlier in the care continuum.^1,3–6^ This strategy, together with advances in resuscitation and surgical strategies, protective equipment, and medical predeployment training, has led to a decrease in case fatality rates by nearly half among United States (US) combat casualties during the recent conflicts in Afghanistan and Iraq compared with the case fatality rate during the Vietnam War.^1–4^

**Box 1. NATO definitions of levels of care in the military medical support chain.**

The military medical support chain is organized in progressive levels of care. A higher level of care holds more capabilities and resources. Casualties receive continuous medical care throughout the medical support chain; however, they do not necessarily pass through each level of care.

**Basic level:**

Self-aid or buddy aid, including care by nonmedical personnel with additional medical education and training. This includes care under fire (POI/hot zone) or care under threat (warm zone).

**Evacuation/en-route care^1,2^:**

MEDEVAC: An organized procedure of transport of patients under continuous medical supervision to or between medical treatment facilities (MTFs). This can either be (1) from POI to the first, most appropriate MTF (forward MEDEVAC), (2) between different MTFs within the Joint Operational Area, usually from a lower to a higher level of care (tactical

MEDEVAC), or (3) transport to an MTF in the home nation or other safe area out of theater (strategic MEDEVAC).

CASEVAC: Unplanned or occasional transport of casualties from POI to an MTF in a nondesignated vehicle without a medical escort. There is limited or no equipment on board to provide en-route medical care. Hence, CASEVAC should not be regarded as a medical capability.

**MTFs^1^:**

**Role 1 MTF:** Provides primary health care, specialized first aid, triage, resuscitation and stabilization, and essential diagnostics. Role 1 MTFs may also include a limited patient holding capability and medical supply capability. Patients are treated for acute minor illnesses or prepared for evacuation to a higher level of care.

**Role 2 MTF:** Provides subsequent damage control resuscitation (DCR) and advanced trauma management including damage control surgery (DCS).

Role 2 Basic (R2B) MTFs can deliver DCS and life, limb, and function saving surgical procedures. They include essential diagnostics, such as field laboratory and basic imaging capabilities, and a limited holding capability.

Role 2 Enhanced (R2E) MTFs are enhanced with varying additional capabilities, including capabilities to stabilize and prepare casualties for strategic evacuation.

Role 2 Forward (R2F) MTFs are mobile and deployable into remote environments to enable advanced resuscitative and surgical treatment as close to the POI as possible. R2F MTFs can also be deployed to support other medical capabilities in theater.

**Role 3 MTF:** Provides specialized health care and surgery, advanced diagnostic imaging (including computed tomography), intensive care units, and postoperative treatment.

**Role 4/5 MTF:** Provides the full spectrum of definitive medical care out of theater, including highly specialized capabilities, such as reconstructive surgery, prosthetics, and rehabilitation. This care is often provided in the casualty’s home nation.

The Role 5 MTFs include major military centers in the United States and provide definitive treatment and rehabilitative care for US service members.^3^

REFERENCES

1. NATO Standardization Office. AJP-4.10, Edition C, Version 1—Allied Joint Medical Support Doctrine. North Atlantic Treaty Organization. Released September 11, 2019. Available at: https://nso.nato.int/nso/nsdd/main/list-promulg

2. NATO Standardization Office. AJMedP-2, Edition A, Version 1—Allied Joint Medical Doctrine for Medical Evacuation. North Atlantic Treaty Organization. Released August 29, 2018. Available at: https://nso.nato.int/nso/nsdd/main/listpromulg.

3. Bagg MR, Covey DC, Powell 4th ET. Levels of medical care in the global war on terrorism. J Am Acad Orthop Surg. 2006;14(10 Spec No.):S7–9.

Analyses of combat casualties from North Atlantic Treaty Organization (NATO) coalition partners during the International Security Assistance Force mission and Operation Enduring Freedom in Afghanistan and during Operation Iraqi Freedom revealed that the majority of deaths occur before reaching a medical treatment facility (MTF).^1,7–10^ In these pre-MTF fatalities, hemorrhage has been identified as the primary cause of potentially preventable deaths.^7^ In addition, in the casualties who died after reaching an MTF, also referred to as “died of wounds,” hemorrhage and its physiologic consequences were also the most common mechanism of death from these potentially survivable injuries.^11^ These findings emphasize the importance of effective bleeding control as soon as possible after injury.

Mortality from extremity hemorrhage has significantly decreased after the full implementation of modern extremity tourniquets in US forces in 2007,^7^ leaving truncal and junctional hemorrhage as the most common causes of potentially survivable deaths in combat casualties.^7,11^ The fatality of these injuries is mainly attributable to their noncompressible or difficult to compress anatomic areas, thereby demanding advanced bleeding control (ABC) techniques at the POI.

Several ABC modalities are available for the management of severe traumatic noncompressible truncal and junctional hemorrhage in combat zones. These include abdominal and junctional tourniquets, wound clamps, Foley catheter balloon tamponade, hemostatic gauzes, injectable hemostatic agents, intra-abdominal self-expanding foam, external pelvic stabilizers, and resuscitative endovascular balloon occlusion of the aorta (REBOA).^12^ The main principle of these tools is to achieve temporary bleeding control to facilitate transport from POI to an MTF with surgical capability. By this, ABC modalities have the potential to improve the outcome of severely injured casualties with noncompressible truncal and junctional hemorrhage. However, several of these modalities are not yet widely adopted and the optimal deployment in combat casualty care (CCC) has not been established.

Following the historic success of the extremity tourniquets, the use and implementation of ABC modalities in CCC should be optimized in an attempt to reduce mortality from noncompressible truncal and junctional hemorrhage. The primary aim of this study was to establish consensus on the optimal deployment of available ABC modalities in CCC, and, secondly, to identify current knowledge gaps and potential areas for improvement regarding current bleeding control guidelines.

## MATERIALS AND METHODS

This study complies with the “Standards for Reporting Qualitative Research” guidelines (Supplemental Digital Content, http://links.lww.com/TA/C302).^13^ An international three-round Delphi survey was conducted using the online survey platform SurveyMonkey. The Delphi technique is a well-established and widely used method that attempts to achieve consensus among experts on a specific topic by using a series of questionnaires.^14^

### Expert Panel

Military physicians with expertise in ABC, clinical experience in deployment in conflict areas, and a high exposure to combat injuries were identified by the investigators. Participants were invited by email, receiving a personal link to the online survey. The level of expertise in ABC of contributing panelists was confirmed in the questionnaire (Supplemental Digital Content 1, http://links.lww.com/TA/C301). The panelists were also asked to identify further colleagues with expertise on this topic. The proposed colleagues were then invited to participate in the expert panel.

### Consensus Definition

Consensus on a topic was reached with 70% or greater agreement and a response rate of at least 70%. The consensus thresholds were defined prior to the data collection and analyses.

### Delphi Survey Rounds

All three phases of the Delphi process consisted of a web-based questionnaire (Supplemental Digital Content 1, http://links.lww.com/TA/C301). The questionnaire focused on: bleeding control modalities, their availability at the various levels of care, potential indications, providers, training requirements, clinical practice guidelines (CPGs), and registries. In-depth attention was placed on REBOA, since this is a rapidly evolving ABC adjunct.

The first round of the Delphi process consisted of a semistructured questionnaire based on previous literature and experiences of the researchers. This well-accepted modified Delphi approach was chosen to compile a complete overview of current practices, to allow elaboration, and to identify issues to be addressed in the second round. The results of the first round were analyzed by the investigators and converted into a structured questionnaire for the second round of the process. This second round consisted of 36 structured questions to further clarify and specify the responses of the panel, and to identify areas of agreement and disagreement. The third and final round consisted of 15 structured questions and was created based upon the results of round 2 where the consensus threshold was not reached. Only the topics where consensus was within reach were addressed in the third round, since only a slight increase in the degree of consensus can be expected between rounds.^14^ The experts received feedback on the questions where no consensus was reached and were asked to revise their judgment or to specify the reasons for remaining outside the consensus. Nonresponders in both rounds 1 and 2 were excluded from the final round. The Delphi process was considered complete when positive or negative consensus (≥70% disagreement on a topic) was achieved or ultimately after three rounds.

### Statistical Analysis

All responses were registered and analyzed in an Excel file (Microsoft Office Excel, Version 12.0; Microsoft Corporation, Redmond, WA) provided by SurveyMonkey. Descriptive statistics were used to present the data by frequencies (percentage).

## RESULTS

In round 1, 70.7% (29/41) of the panel members responded to the questionnaire. Four of the invited panel members declined to participate. Three invited panelists opted out after round 1 and 27 panel members responded to the second round of the survey (27/38, 71.1%). In the final round, 28 panel members responded (28/31, 90.3%) to the survey. Nonresponders in both rounds were excluded (n = 7). In total, 32 panel members from 10 different nations and with varying medical specialties (Table 1) completed at least one survey round. Twenty-seven panel members (27/29, 93.1%) responded to the question regarding their expertise in ABC. They were all experts in ABC (27/27, 100%).

**TABLE 1:** Nationality and Medical Specialty of the Expert Panel Members

Nationality	n	%
American	11	34.4
British	6	18.8
Canadian	1	3.1
Danish	1	3.1
Dutch	2	6.3
French	4	12.5
German	3	9.4
Israeli	2	6.3
Norwegian	1	3.1
Swedish	1	3.1
**Medical Specialty**	**n**	**%**
Surgery	24	75
Trauma, acute care, or vascular surgery	23	71.9
Surgery other	1	3.1
Emergency medicine	6	18.8
Interventional cardiology	1	3.1
Anesthesiology	1	3.1
N_Total_	32	

### Bleeding Control Toolbox

With regard to the current availability of ABC modalities, 65.5% (19/29) of the panel members responded that their military system has junctional tourniquets available. The availability of wound clamps was 28.6% (8/29), intra-abdominal gas insufflation 0% (0/29), intra-abdominal self-expanding foam 7.1% (2/29), hemostatic agents 93.1% (27/29), pelvic stabilizers 96.6% (28/29), and REBOA 75.9% (22/29). There was a great variety in the available types or brands of the various bleeding control modalities, except for the wound clamp. This was the iTClamp in all cases.

The expert panel has reached consensus that a standard toolbox for bleeding control in (austere) military environments should at least include bandages, junctional and limb tourniquets, pelvic binders/stabilizers, and hemostatic agents. The panel also reached consensus that, for trained personnel, REBOA should be part of this standard bleeding control toolbox (Table 2). Remarks from panel members included that the medical team performing REBOA should be extensively trained and that there should be access to surgical care within a limited timeframe of less than 45 minutes. No consensus has been reached on whether there is any indication for the use of a wound clamp in (austere) military environments (14/28, 50%) and whether it should be part of the standard toolbox. Arguments against its use were that there is no robust clinical evidence of efficacy to justify replacement of existing available options. Arguments for its use were that it is easily applied, that it might be a useful tool for less skilled providers, that it may offer speed advantages in prehospital care or in multiple-injured casualties, and that it can be beneficial for specific injuries, such as craniomaxillofacial wounds.

**TABLE 2:** Overview of Expert Panel Responses Regarding the Standard Bleeding Control Toolbox, the Availability of Bleeding Control Modalities at the Different Levels of Care, and Providers of the Bleeding Control Modalities

The Standard Toolbox for Bleeding Control in (Austere) Military Environments:	Panel Members Agreeing (n)	Consensus Reached
(1) Should at least include bandages, junctional and limb tourniquets, pelvic binders/stabilizers and hemostatic agents	25/27; 92.6%	Yes
(2) Should include REBOA, for trained personnel	21/28; 75.0%	Yes
(3) Should include a wound clamp	12/27; 44.4%	No
(4) Should include abdominal gas insufflation	0/27; 0%	Yes*
(5) Should include intra-abdominal self-expanding foam	4/27; 14.8%	Yes*

Availability of resources:	Panel members agreeing (n)	Consensus reached
(6) The standard toolbox for bleeding control should be available at all levels of care (from POI to role 3 facilities)**	27/28; 96.4%	Yes
(7) REBOA should be available at the POI/warm zone†	19/27; 70.4%	Yes
(8) REBOA should be available at the casualty collection point near the combat zone†	21/27; 77.8%	Yes
(9) REBOA should be available during en-route care†	23/27; 85.2%	Yes
(10) REBOA should be available in role 1 MTFs or forward surgical hospitals‡	24/26; 92.3%	Yes
(11) REBOA should be available in fixed Role 2/3 MTFs‡	25/26; 96.2%	Yes

Providers:	Panel members agreeing (n)	Consensus reached
(12) Invasive bleeding control modalities should only be applied by trained physicians	22/28; 78.6%	Yes
(13) Medics should be allowed to apply invasive bleeding control modalities	7/28; 25.0%	Yes*
(14) All medical personnel should be allowed to apply noninvasive bleeding control modalities	27/28; 96.4%	Yes
(15) Both medical and nonmedical personnel should be allowed to apply noninvasive bleeding control modalities	27/28; 96.4%	Yes

*Negative consensus was reached.

**Provided that there are protocols when and by whom to use the various modalities.

†Considering that adequate training conditions are met and the casualty can be transported into an OR within 45 minutes with a dedicated MEDEVAC.

‡Considering that adequate training conditions are met.

### Availability of Resources

Not every bleeding control tool is currently available at all levels of care, and the availability at the different levels of care varies between military systems. The expert panel reached consensus that the standard toolbox for bleeding control should be available at all levels of care (from POI to role 3 facilities), provided that there are protocols when and by whom the use of the various modalities is indicated (26/27, 96.3%). Consensus has also been reached that REBOA should be available at all levels of care (Table 2), considering that adequate training conditions are met and casualties can be transported to an operating room (OR) within 45 minutes with a dedicated MEDEVAC (if applicable). Opponents of this statement for REBOA at the POI/warm zone (29.6%) argued that the casualty should be transported to an OR within 30 minutes, that surgical treatment must be immediate to avoid overwhelming reperfusion injury, that REBOA may cause death if placed in the wrong patient, and that there is currently not enough evidence to support this or recommend it formally. Arguments of the panelists that advocate the availability of REBOA at the POI, considering the specific conditions (training, time to OR, MEDEVAC), were that data show that trauma patients who die from hemorrhage are more likely to die in the first 20 minutes to 30 minutes after injury, thus the closer a skilled team with the full range of bleeding control tools is, the better the survival opportunities for a patient are, that REBOA should be used directly in the combat zone when the area is safe, that there are FDA-approved REBOA devices that have been shown safe and feasible by small military surgical teams in austere locations, and that REBOA should be implemented once appropriately trained and in accordance with Joint Trauma System (JTS) and similar guidelines.

### Providers and Training

The expert panel reached consensus that invasive bleeding control modalities, such as REBOA or intra-abdominal foam or gas, should only be applied by trained physicians, but that all medical and nonmedical personnel should be allowed to apply noninvasive bleeding control modalities (bandages, junctional and limb tourniquets, pelvic binders/stabilizers, and hemostatic agents) (Table 2).

To train providers in the use of bleeding control modalities, most nations use a training course specifically designed by their military service (23/27, 85.2%), combined with a variety of general battlefield and/or civilian trauma courses. Most systems have an additional course to prepare providers specifically for REBOA use (18/26, 69.2%) or have REBOA training integrated in the general bleeding control course (3/26, 11.5%). The experts agreed that, for adequate preparation of military care providers, a training curriculum for ABC modalities should include all of the following: a didactic component, simulator skills, animal laboratory skills and cadaver skills. They also agreed that endovascular bleeding control skills should be a standard part of the training curriculum for military care providers (Table 3).

**TABLE 3:** Overview of Expert Panel Responses Regarding the Training of Bleeding Control Providers, Registries, and Guidelines

Training:	Panel Members Agreeing (n)	Consensus Reached
(1) A training curriculum for ABC modalities should include all of the following: a didactic component, simulator skills, animal laboratory skills and cadaver skills	23/27; 85.2%	Yes
(2) Endovascular bleeding control skills should be a standard part of the training curriculum for military care providers	23/27; 85.2%	Yes
(3) There should be an official guideline dictating the frequency of ABC training	25/27; 92.6%	Yes
(4) Providing physicians should follow refresher training for ABC skills in general at least every 2 years and before deployment	22/28; 78.6%	Yes
(5) Training of endovascular bleeding control skills should be refreshed more frequently than other bleeding control skills training	20/27; 74.1%	Yes
(6) Providing physicians should follow refresher training for endovascular bleeding control skills at least annually and before any deployment	24/28; 85.7%	Yes
(7) Providing nonphysicians should follow refresher training for ABC skills at least annually and before any deployment	26/28; 92.9%	Yes

Registries and guidelines:	Panel members agreeing (n)	Consensus reached
(8) There should be an international collaboration to formulate best CPGs and recommendations on bleeding control care	19/27; 70.4%	Yes
(9) In addition to an international CPG dictating bleeding control care, each nation should be able to make its own nation-specific adjustments	26/28; 92.9%	Yes
(10) In a formal CPG dictating bleeding control care, REBOA should be explicitly discussed	27/27; 100%	Yes
(11) There should be an international collaboration to register patients in whom ABC devices are deployed in an international registry	20/27; 74.1%	Yes
(12) There should be an international collaboration to collect data on bleeding control to capture “lessons learned” or for process improvement	19/27; 70.4%	Yes
(13) There should be an international collaboration to capture data on REBOA use other than the patient case history to capture “lessons learned” or for process improvement	23/27; 85.2%	Yes

Repetition of training for ABC skills varied between the military systems and formal policies often do not exist (10/26, 38.5%). The experts agreed that there should be an official guideline dictating the frequency of training such skills to limit or prevent degradation of skills after initial training. Consensus has also been reached on the preferred frequency of refresher training (Table 3).

### Guidelines and Registries

The majority of participating experts indicated that their system has one or more formal CPGs dictating bleeding control care (24/27, 88.9%) and 17/26 (65.4%) have a CPG dictating REBOA care. The expert panel agreed that there should be an international collaboration to formulate best CPGs and recommendations for bleeding control care, and that REBOA should be explicitly discussed in such a CPG (Table 3).

Fourteen respondents replied that their military system has a registry to record patients in whom advanced bleeding devices are deployed (14/27, 51.9%), and 10 of 27 (37.0%) other panelists responded that they are interested in such a registry. Consensus was reached that there should be an international collaboration to record these patients in an international registry (20/27, 74.1%).

In addition, 66.7% (18/27) of the panel members responded that their system has a formal process to collect data on bleeding control procedures, other than the patient case history, to capture “lessons learned” or for process improvement. There were 34.6% (9/26) that responded that their system has such a process to collect specific data on REBOA use. Again, the expert panel agreed that there should be an international collaboration to record these bleeding control procedures and REBOA data to evaluate and improve CCC (Table 3).

### REBOA

The preferred vascular access site for REBOA was the common femoral artery (22/23, 95.7%) and the preferred means of arterial access was ultrasound-guided (19/24, 79.2%). There was no consensus on the preferred means of vascular access site closure: open surgical repair 18.5% (5/27), manual compression 37.0% (10/27), manufactured pressure device 11.1% (3/27), manufactured closure device 0% (0/27), depends on the situation 33.3% (9/27). The panel agreed that a guidewire-free device should be used for REBOA when it is used outside a surgical facility and if there is no fluoroscopic guidance available (25/27, 92.6%). Regarding fluoroscopy-free REBOA, recent literature describes a significant variance in aorta zone 3 depths, making the use of anatomical landmarks (level of umbilicus) of increased risk of malpositioning,^15^ but describes a 100% correlation between mid-sternum and zone 1.^16^ Given this knowledge, the expert panel was asked whether they agreed with the statement to use REBOA in aorta zone 1 (between the left subclavian artery and celiac trunk) in a fluoroscopy-free environment. Nineteen of 28 experts agreed with this statement (67.9%).

The panel members have reached consensus that REBOA is indicated in military environments, assuming that surgical care will be available within an acceptable timeframe, in hemodynamic unstable patients with junctional groin injuries, pelvic injuries, or traumatic cardiac arrest (Table 4). In addition, 85.7% (18/21) responded that in their system, REBOA is used for penetrating abdominal injuries, and 81.0% (17/21) responded that it is used for blunt abdominal injuries. The panel members also agreed that in military environments, REBOA is not contraindicated in hemodynamic unstable patients with multiple major bleeding sites, again assuming that surgical care will be available within an acceptable timeframe. The use of REBOA for solitary major neck injuries was considered contraindicated. No consensus was reached on the use of REBOA in military environments in hemodynamic unstable patients with chest injuries, junctional axillary injuries, or a major neck injury in combination with one or more major thoracoabdominal bleeding sites (Table 4). Suggested indications by the panelists for REBOA in patients with chest trauma were as follows: patients in pericardiac arrest (zone 1 REBOA), unstable patients without surgical resources to treat penetrating chest injuries, patients with thoracoabdominal injuries, combined use of REBOA and a median sternotomy, and for every injury in dying patients after exclusion of relevant bleeding in the thorax by bilateral thorax drainage. Panelists also stated that REBOA should be considered if chest decompression has relieved potential pneumothorax and point-of-care ultrasound has excluded pericardial tamponade, and that thoracic injuries without massive hemorrhage should not be contraindications for REBOA.

**TABLE 4:** Current Indications for Which REBOA Is Used Among the Military Systems and Expert Panel Responses Regarding the Indications and Contraindications for REBOA Use in Military Environments

Current Use of REBOA Among Military Systems	Panel Members Confirming (n)	
Neck injury	1/21; 4.8%	
Junctional injury	15/21; 71.4%	
Penetrating thoracic injury	3/21; 14.3%	
Blunt thoracic injury	4/21; 19.1%	
Penetrating abdominal injury	18/21; 85.7%	
Blunt abdominal injury	17/21; 81.0%	
Multiple bleeding sites	7/21; 33.3%	
Traumatic cardiac arrest	11/21; 52.4%	
Other: all necessary injuries/no differentiation specified	2/21; 9.5%	

Expert panel consensus	Panel members agreeing (n)	Consensus reached
Use of REBOA indicated in military environments*		
Junctional groin injury	25/27; 92.6%	Yes
Junctional axillary injury	14/26; 53.9%	No
Pelvic injury	26/27; 96.3%	Yes
Traumatic cardiac arrest	20/27; 74.1%	Yes
Use of REBOA not contraindicated in military environments*		
Multiple major bleeding sites	24/27; 88.9%	Yes
Use of REBOA contraindicated in military environments		
Solitary major neck injury	23/27; 85.2%	Yes
≥1 major thoracoabdominal bleeding sites *and* a major neck injury	16/27; 59.3%	No
≥1 major bleeding site below the diaphragm *and* a major neck injury	18/27; 66.7%	No
Blunt thoracic injury	17/27; 63.0%	No
Penetrating chest injury	16/27; 59.3%	No

*Among hemodynamic unstable patients and assuming that surgical care will be available within an acceptable timeframe.

## DISCUSSION

Optimizing the deployment of ABC modalities in CCC is essential to improve the survival from noncompressible truncal and junctional hemorrhage. This study has established consensus among an expert panel of military physicians on topics regarding the contents of a standard bleeding control toolbox in (austere) military environments, the availability of ABC modalities at the different levels of care, the providers that should be allowed to apply invasive and noninvasive bleeding control modalities, and training requirements. Consensus was also reached on the necessity of international registries and guidelines, and on certain indications and contraindications for REBOA in CCC. Furthermore, this study highlights the demand for clinical data on bleeding control modalities, such as the wound clamp and abdominal foam, and for clarity on the benefit of REBOA for patients with chest injuries, axillary hemorrhage, and truncal hemorrhage combined with neck injuries.

Care providers in combat environments have to operate under extraordinary circumstances. Areas can be potentially hostile, resources are limited, and there are often multiple casualties requiring care at the same time.^6^ In these stressful situations, where minutes can matter, it is of great importance to be familiar with the available equipment. Standardization of the available resources in types and brands, for instance, with a standardized bleeding control toolbox, may contribute to an improvement of the efficiency of the providing team. This may be particularly beneficial when providers from different teams or coalition partners are cooperating. The expert panel has reached consensus on the minimum contents of a standard bleeding control toolbox and that it should be available at all levels of care, from POI to role 3 MTFs. Naturally, provided that adequate training conditions for the providers are met and that there are protocols when and by whom to use the various modalities. An example of such a protocol is the US JTS Tactical Combat Casualty Care Quick Reference Guide.^17^

Several nations have CPGs for CCC available and some nations use available CPGs of other nations. The extent of elaboration on bleeding control care varies between different guidelines. Of the open-source CPGs, the US JTS-CPGs are the most comprehensive. This study reveals the desire for an international collaboration to formulate best CPGs for bleeding control care. In addition, the experts agreed that there should be guidelines dictating bleeding control training requirements, involving a higher frequency for endovascular skills than for other bleeding control skills, and a higher frequency for nonphysicians than for physicians. It would be interesting to explore whether nonphysicians also feel the need for more frequent refresher training. Degradation of skills studies could confirm the appropriate training frequency in both populations.

This study also highlights the need for more profound data on the relatively new bleeding control adjuncts, such as the wound clamp. Although 50% of the experts responded that there is no indication for a wound clamp in (austere) military environments, the iTClamp is recommended in multiple JTS-CPG guidelines for external hemorrhage of the head and neck or to improve the effectiveness of wound packing.^18,19^ Detailed case registration can contribute to increasing the scientific basis and recommendations for the use of these adjuncts.

Several nations have developed a trauma registry to describe, evaluate, and improve casualty care.^20,21^ The value of these trauma registries on battlefield outcomes has previously been described.^20,22,23^ However, prehospital data are often not fully documented, while the majority of fatalities occur in this domain.^4,5,20,21,24,25^ Hence, essential information might be missing from current analyses. Furthermore, as the current study demonstrates, specific data on bleeding control measures for truncal and junctional hemorrhage is often not extensively captured in present registries.^20,21^ The JTS does have a process to collect comprehensive REBOA data for process improvement, but data on other ABC modalities are not registered in such detail.^4,26^ The expert panel agreed that there should be an international collaboration to record these data, both for patient case history and for process improvement, and to capture lessons learned. This supports the previous recommendation of Van Dongen et al.^21^ to implement a United Nations/NATO wide registry system for further improvement of CCC and the registration of casualties. The concept of an international registry has already been proven feasible in a civilian setting.^27^ The benefit of an international registry is that smaller nations with a low casualty case load will be able to participate. Furthermore, pooling casualty cases might generate potentially valuable information for trauma care improvement. Of course, national legislations of contributing partners and handling of classified information are issues that have to be addressed in such an international collaboration.

An international registry capturing bleeding control data should at least include specific data on the bleeding control modalities used, type of injuries treated, where and by whom the adjuncts were used, the effectiveness of the modalities, complications, and experiences from the providers. These variables are needed to generate profound data on the various bleeding control modalities and their potential role in combat trauma systems and should, therefore, be collected throughout the entire care continuum, starting at the POI. Although collection of prehospital data might be challenging, it has been proven feasible. It does, however, require a continuous effort to improve the collection process and encourage providers to enter the data.^20,28^

More high-grade evidence is also needed on indications for REBOA in austere environments. Resuscitative endovascular balloon occlusion of the aorta is currently used in both civilian hospital and prehospital settings^29^ and in select military medical units. Under the EndoVascular Hybrid Trauma and bleeding Management concept, it might be a valuable tool in military environments,^30,31^ even in prehospital settings in patients who otherwise bleed to death before reaching a surgical facility.^32^ Current REBOA reports concern predominantly civilian cases, while both circumstances and injury patterns are different in military environments. During the recent conflicts in Afghanistan and Iraq, the most common mechanisms of injury were blast injuries, often resulting in multisystem, combined blast, blunt, and penetrating injuries.^1,33^ However, current JTS guidelines do not discuss REBOA in case of multicavitary injuries, while the expert panel agreed that REBOA is not contraindicated in patients with multiple major bleeding sites. Also, resources are limited in military environments and time to definitive surgical treatment may be prolonged. There was a consensus among the expert panel that REBOA should be available at all levels of care, including the prehospital arena. This statement is supported by recent gap analyses and case series on REBOA in austere environments.^34–38^ Modern REBOA devices are specifically developed for trauma cases and carry features that make them suitable for application in austere, resource-limited environments.^39^ However, techniques for lengthening balloon occlusion time have to be explored given the increasing shift to prolonged field care or for cases where the number of casualties exceeds the surgical capacity.^30,40^ Currently evolving strategies to lengthen occlusion time by mitigating ischemia-reperfusion complications are intermittent balloon inflation and partial balloon inflation to allow some distal flow. However, several studies showed that it is difficult to titrate partial REBOA with current REBOA catheters.^41,42^ Recently, a novel REBOA device specifically designed for partial REBOA that eliminates the need for frequent balloon titration has been released and is currently being applied in select trauma centers in the United States and Canada.^43^

There are limitations to this study. Not all experts responded to every survey round, and it might be possible that not all experts on the topic were invited. To reduce the chance of missing key experts, all invited panel members could nominate colleagues for the panel. The military nature of this study may have influenced expert responses, particularly in the exploratory first round with a relatively high rate of skipped questions. As this study highlights, current evidence to support the consensus statements is limited. Further research should, therefore, be encouraged. Furthermore, a Delphi method cannot forecast future developments. Hence, new scientific developments may alter expert opinions. Lastly, three panelists were also involved in the writing of this article. They are all experienced in military trauma surgery with a high exposure. Data analysis was performed by other investigators to exclude personal experience from the analysis.

## CONCLUSION

This study has established consensus among an expert panel of military physicians on topics regarding the contents of a standard bleeding control toolbox, the availability of ABC modalities at the different levels of care, and the providers that should be allowed to apply invasive and noninvasive bleeding control modalities. Furthermore, consensus was reached on the necessity of international registries and guidelines, and on certain indications and contraindications for REBOA in CCC. These results can be used to optimize the care for seriously injured combat casualties and improve the chance of survival. More clinical data are needed on the wound clamp, abdominal foam, and the benefit of REBOA for patients with chest injuries, axillary hemorrhage, and truncal hemorrhage combined with neck injuries.
